# Extracting quantitative genetic interaction phenotypes from matrix combinatorial RNAi

**DOI:** 10.1186/1471-2105-12-342

**Published:** 2011-08-17

**Authors:** Elin Axelsson, Thomas Sandmann, Thomas Horn, Michael Boutros, Wolfgang Huber, Bernd Fischer

**Affiliations:** 1 EMBL European Bioinformatics Institute, Wellcome Trust Genome Campus, Hinxton, Cambridge CB10 1SD, UK; 2 Genome Biology Unit, EMBL, Meyerhofstraße 1, D-69117 Heidelberg, Germany; 3 German Cancer Research Center (DKFZ), Div. Signaling and Functional Genomics and Department of Cell and Molecular Biology, Faculty of Medicine Mannheim, Heidelberg University, Im Neuenheimer Feld 580, D-69120 Heidelberg, Germany; 4 CellNetworks Cluster of Excellence, Heidelberg University, Im Neuenheimer Feld 267, D-69120 Heidelberg, Germany; 5 Hartmut Hoffmann-Berling International Graduate School (HBIGS), Heidelberg University, Im Neuenheimer Feld 501, D-69120 Heidelberg, Germany

## Abstract

**Background:**

Systematic measurement of genetic interactions by combinatorial RNAi (co-RNAi) is a powerful tool for mapping functional modules and discovering components. It also provides insights into the role of epistasis on the way from genotype to phenotype. The interpretation of co-RNAi data requires computational and statistical analysis in order to detect interactions reliably and sensitively.

**Results:**

We present a comprehensive approach to the analysis of univariate phenotype measurements, such as cell growth. The method is based on a quantitative model and is demonstrated on two example Drosophila cell culture data sets. We discuss adjustments for technical variability, data quality assessment, model parameter fitting and fit diagnostics, choice of scale, and assessment of statistical significance.

**Conclusions:**

As a result, we obtain quantitative genetic interactions and interaction networks reflecting known biological relationships between target genes. The reliable extraction of presence, absence, and strength of interactions provides insights into molecular mechanisms.

## Background

Population variations in complex phenotypes, including many human diseases, are not caused by single polymorphisms, but result from variations in multiple genes as well as from environmental factors [1]. The effect of multiple genetic polymorphisms is typically not additive [2], but can interact in complex ways. An understanding of this *pervasive epistasis *will facilitate a better understanding of the molecular mechanisms underlying phenotypes. The use of genetic interactions has also been proposed to allow more specific drugs with less side-effects [3].

While, in general, phenotypic variation may be caused by interactions of any number of genes and environmental factors, important insights may already be gained from understanding pairwise gene-gene interactions. In many cases, pairwise interactions can provide clues for the placement of gene products in molecular interaction networks [4]. Furthermore, the quantitative interaction profile of one gene with many other genes can itself be considered a multivariate phenotype of that gene, and is a powerful indicator of its molecular function [5-9].

In yeast, deletion-mutant libraries have allowed large-scale automated analysis of double mutants using methods like SGA [6,10], SLAM [11], dSLAM [12], E-MAPS [13] and GIM [14]. In worms, genetic interactions have been discovered by visual scoring of RNAi applied to genetic mutants [15] or by pairwise combinatorial RNAi [16]. In cell lines from higher organisms, including Drosophila and human, genome-wide RNAi screens have been successful in identifying single-gene effects [17]. Recently, large-scale combinatorial RNAi experiments have been used to map genetic interactions in Drosophila cells [18]. Many laboratories are now working on similar combinatorial RNAi experiments, and effective methods for analysis of the data are needed.

We will now introduce the required concepts. A quantitative phenotype *y *can be modelled as a function *f *of genetic and environmental factors *x*_1_,..., *x_n_*:

(1) $$y=f\left(x_{1},\;\ldots ,\;x_{n}\right).$$

The phenotype *y *can, for example, be a measure of the growth cells, but more general situations may be considered.

Now consider a particular genetic and environmental background $x_{1}^{0},\;\ldots ,\;x_{n}^{0}$; often, this is called wild-type under standard laboratory conditions. We denote the resulting phenotype by *y*^0 ^and approximate the phenotypic variations around *y*^0 ^that are caused by differences from $x_{1}^{0},\;\ldots ,\;x_{n}^{0}$ by analytic expansion [19]

(2) $$\begin{array}{lll} y-y^{0}= & \overset{n}{\underset{i=1}{\sum }}m_{i}\left(x_{i}-x_{i}^{0}\right)+\; & \text{(1)}\\ & \;\frac{1}{2}\overset{n}{\underset{i,j=1}{\sum }}w_{ij}\left(x_{i}-x_{i}^{0}\right)\left(x_{j}-x_{j}^{0}\right)+\ldots ,\; & \text{(2)}\\ & \; & \text{(3)} \end{array}$$

where the coefficients *m_i _*represent the effects of single-gene or single-factor perturbations, *w_ij _*are pairwise interaction coefficients and ... stands for higher order terms. In an additive situation, the higher order terms as well as the quadratic term involving the pairwise interaction coefficients *w_ij _*are negligible, and the phenotype variations *y *- *y*^0 ^are sufficiently well explained by the linear term, i. e. the first sum on the right hand side of Equation (2). We say that epistasis is present, or equivalently, that an interaction is present, if any of the second or higher order terms plays a significant role. There is ample evidence for epistasis in many phenotypes of interest [2,20-22].

The choice of scale on which the phenotype *y *is modelled is important (for example, whether or not measurements are logarithm-transformed; [23]), and alternative definitions of what should be called an interaction have been considered [24]. We will return to these questions.

## Results and Discussion

### Data set

To develop experimental and computational methods, we produced a benchmark data set from cultured *Drosophila melanogaster *cells (S2 cells). The phenotypic readout, after five days of co-RNAi incubation of cells in 384-well plates, was total ATP content, which served as a measure of overall cell viability [25].

All pairwise interactions between 16 different genes were assayed: 8 genes with a previously characterised role in cell-cycle regulation and 8 genes selected randomly from the transcriptome by use of a computer random number generator. The 8 random genes happened to contain a few well-known multifunctional genes, including the cell cycle regulator Rbf. Gene ontology (GO) annotation terms for the 16 genes are provided in Additional File 1: Table S**1**.

The cells were incubated with all 16 _* _15/2 = 120 pairwise combinations of dsRNAs targeting these genes. The experiment was performed with two biological replicates, using different passages of the cells; each of these contained 10 technical replicates. Hence, the data set consists of 20 measurements for each dsRNA combination and 2,400 measurements in total.

We adapted a *criss-cross *design [26]. To this end, two different 384-well stock plates were prepared: one *row plate*, where each of the single dsRNAs occupied a full row of wells, and one *column plate*, where each of the dsRNAs was placed into a full column. By combining reagents from the row plate with those from the column plate, each pairwise combination of dsRNAs was obtained twice. For each of the two biological replicates, five plates were incubated and analysed.

### Adjustment for plate effects and quality assessment

We applied plate normalisation [27]

(3) $$y_{pi}=y_{pi}^{pre}-{\hat{\mu }}_{p},$$

where $y_{pi}^{pre}$ was the logarithm-transformed (base 2) luminescence intensity of the *i*-th well in plate *p *and ${\hat{\mu }}_{p}$ was a plate-specific correction coefficient. Methods for computing ${\hat{\mu }}_{p}$ from the data need to be adapted to the experiment [28]. Here, we used the midpoint of the shorth of all values from plate *p *for wells with co-RNAi, but not from control (Additional File 2: Figure S1). The shorth of a distribution is the shortest interval that contains half of the data; its midpoint can serve as an estimator of the mode of the distribution, and the estimate is generally less affected than, e.g., mean or median by skewness or outliers in the data. Diagnostic plots for quality assessment showed no significant spatial artifacts (Additional File 3: Figure S2). Reproducibility was assessed by scatter plots between replicates (Additional File 4: Figure S3). In Section **Choice of scale and neutrality function**, we provide reasons for the choice of the logarithm transformation for the analysis of this data and discuss alternative approaches.

### Estimating main effects and interactions

Next, we obtained estimates of interaction effects *w_ij _*from the co-RNAi data. First, suppose that the baseline value *y*_0_, the double knock down phenotype *y_ij _*and the single-gene effects *m_i _*are known. Then, for *i *≠ *j*, the interaction term *w_ij _*can simply be obtained from Equation (2) by solving *w_ij _*= *y_ij _*- *y*_0 _- *m_i _*- *m_j_*. Note that third and higher order terms are not present in a pairwise co-RNAi experiment. Now let *y_ijk _*be the *k*-th replicate measurement for the combinatorial knock down of genes *i *and *j*, and suppose that we have estimated ${ŷ}_{0k}$ as the baseline phenotype in replicate *k *and ${\hat{m}}_{ik}$ as the single-gene effect of gene *i *in replicate *k*. We used the data version of the above relationship to motivate the estimator

(4) $${ŵ}_{ij}=\frac{1}{K}\overset{K}{\underset{k=1}{\sum }}\varepsilon_{ijk},\;where$$

(5) $$\varepsilon_{ijk}=y_{ijk}-{ŷ}_{0k}-{\hat{m}}_{ik}-{\hat{m}}_{jk}.$$

To obtain the main effect estimates ${\hat{m}}_{ik}$ and the baseline estimates ${ŷ}_{0k}$, we minimised the sum of squares,

(6) $$\left({ŷ}_{01},\;\ldots ,{ŷ}_{0K},{\hat{m}}_{11},\;\ldots ,{\hat{m}}_{NK}\right)=arg\;min\overset{N}{\underset{i,j=1}{\sum }}{ŵ}_{ij}^{2}.$$

In the rest of this section, we provide the motivations for this approach, contrast it with plausible alternative choices, discuss implementation and describe variations that may be useful for other applications.

#### Identifiability

In order to make the solution of criterion (6) unique, a further condition is necessary, for instance $\sum_{i}{\hat{m}}_{ik}=0$ for each *k*. The choice of this condition does not affect the estimated interactions ${ŵ}_{ij}$ and serves mainly for computational convenience.

#### Parameterisation

In general, equations (5) and (6) allow different values for the baseline ${ŷ}_{0k}$ and the single gene effects ${\hat{m}}_{ik}$ for different replicates *k*. Here, we allowed for different ${ŷ}_{0k}$ and ${\hat{m}}_{ik}$ between the two biological replicates, but set them equal within the 10 technical replicates each. Hence, 32 parameters were fitted, for 16 dsRNAs in 2 biological replicates. We have found it useful to allow different values for the two biological replicates in order to adjust for slight, but detectable variations in experimental parameters such as number of cells seeded, incubation time, dsRNA reagent concentration and transfection efficiency. Allowing the parameters ${ŷ}_{0}$ and *m_i _*to pick up some of this - unintended, but hardly avoidable - variability in the data, we expect to have arrived at better estimates of the interaction effects *w_ij_*. Such an approach is analogous to using different, matched normalisation controls in different parts of an experiment. If we had been confident that across replicates these values were actually the same, then we could have set them to be equal in (5) and (6), thus reducing the number of parameters to 16 and slightly increasing the precision of the estimates. On the other hand, if model fit diagnostics had indicated that allowing these parameters to even be different for each technical replicate would fit the data substantially better, a more highly parameterized model with 320 parameters could have been fit, with a loss in estimation precision.

#### Use of single-gene and non-treated controls

Criterion (6) only uses the data from the co-RNAi wells for the estimation of the baseline and the single gene effects. It does not require, or use, measurements from single gene RNAi treatments or untreated control wells. Our rationale for doing so was as follows. Ideally, the values obtained through minimisation of (6) and from direct measurements in controls should be the same; however, more data points were available for evaluating criterion (6) than there are control measurements, hence the former provided better precision and was more robust against individual outlier data points. Furthermore, if there were a difference between baseline and single gene effect estimates obtained in the two different ways, then we would prefer the estimates from criterion (6), since -by construction- they lead to more conservative estimates of interaction effects. In fact, criterion (6) can be motivated by the assumption that interactions should be rare, and by the aim of explaining as much possible of the observed variation in *y_ijk _*through baseline and single gene effects, and only considering what remains from that as interactions. If available, we propose using the data from single gene and untreated control wells for quality control: compare them to the estimates ${ŷ}_{0k}$ and ${\hat{m}}_{ik}$ obtained from (6), and deviations would indicate an experimental problem that needs to be attended to before further interpretation of the data.

#### Robustness

Instead of minimising the sum of squares (6), a robust variant could be chosen such as minimising the ${ℒ}_{1}$-norm or a trimmed sum of squares (LTS regression; [29]), or using M-estimation [30]. For our data, exploration of these methods did not lead to substantially different results. In other situations, however, such variants may be appropriate, for example, when the proportion of interactions is not small, or when some of them are large, and we advise researchers to check their data for such effects.

### Assessing statistical significance

For each pair of genes (*i*, *j*) we applied the ordinary *t*-statistic to the residuals *ε_ijk _*across the 20 measurements (*k *= 1,..., 20) to weigh the evidence for the interaction to be non-zero. However, the large number of replicates is a peculiarity of our benchmark dataset, and cannot be expected in general. With few replicates, the ordinary *t*-test has two problems: first, due to the small number of degrees of freedom, the test will have little power, leading to a loss of discoveries. Second, it becomes likely that, by chance, small estimates of the variance (the denominator of the *t*-statistic) are obtained even if the true variance is larger, leading to a fraction of discoveries with small effect sizes that would not be replicated if the experiment were repeated. To address both of these problems, in the context of microarray analysis the moderated *t*-test has been proposed [31,32]; we investigated the same approach here.

We wanted to assess the relative performance of three different ranking methods: the *p*-value from the ordinary *t*-test, the *p*-value from the moderated *t*-test, and the average effect ${ŵ}_{ij}$ (that is, simply the numerator of the *t*-statistic). For this, we set up a benchmark set of true interactions (positives) and non-interactions (negatives). We considered those pairs true positives that had, on the full data set with 20 replicates, a nominal *p*-value of less than 0.001 in the ordinary *t*-test; all other pairs were considered true negatives.

To simulate an application setting with few replicates, we applied the ranking methods to the data from a single plate, hosting two technical replicate measurements per gene pair. We applied a set of thresholds, with decreasing stringency, to the three ranking methods and obtained the corresponding hit list. We then, for each hit list, computed the true positive rate (TPR) as the ratio between the number of true (as defined by the reference) hits and the total number of positives, and the false positive rate (FPR) as the ratio between the number of false (as defined by the reference) hits and the total number of negatives. This resulted in one ROC curve per plate.

**Figure 1:**
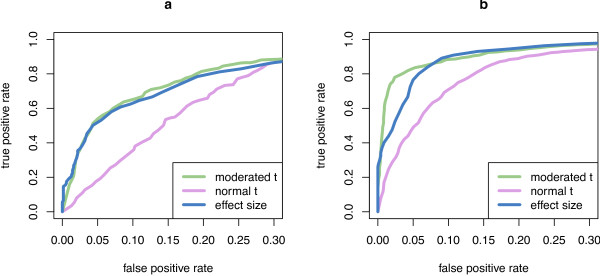
**Comparison of test statistics**. Comparison of test statistics. The plots show average ROC curves for normal *t*-statistic, moderated *t*-statistic and effect size. Panel a shows the results when using data from two technical replicates, panel b is from two biological replicates with two technical replicates each. The moderated *t*-statistic and effect size outperform the ordinary *t*-statistic in both scenarios; on data with biological replicates, the moderated *t*-statistic performs better than the effect size.

Figure 1 indicates clear benefits from using the moderated *t*-test. The ordinary *t*-test generally performs poorly in such situations with limited amount of replication. When only two technical replicates from the same plate were used (panel a), the variance of replicates was constant or close to constant across the different interaction pairs. Hence, the variance estimates used in the moderated *t*-test were the same or almost the same for all gene pairs, making the resulting moderated *t*-statistic proportional -and therefore equivalent- to the numerator of the *t*-statistic (the average effect size ${ŵ}_{ij}$), and no significant difference in performance was seen between the two methods in this setting. However, when variation is higher, as is the case when also biological replicates were considered (Panel b), the moderated *t*-test was able to pick up some of the gene-pair dependence of the variance and outperformed the average effect size ${ŵ}_{ij}$.

The reference that we used for this benchmark, as described above, is not a *ground truth *in a strict sense, and the TPR and FPR values may be biased because of errors in the reference. However, for the purpose of method comparison, the relative positions of the ROC curves of the three ranking approaches are still informative as long as the reference set is enriched for ground truth compared to a random set; in effect, the biases cancel out each other. This pragmatic use of the ROC has also been called *pseudo-ROC *analysis [33].

### Resulting interactions

**Figure 2:**
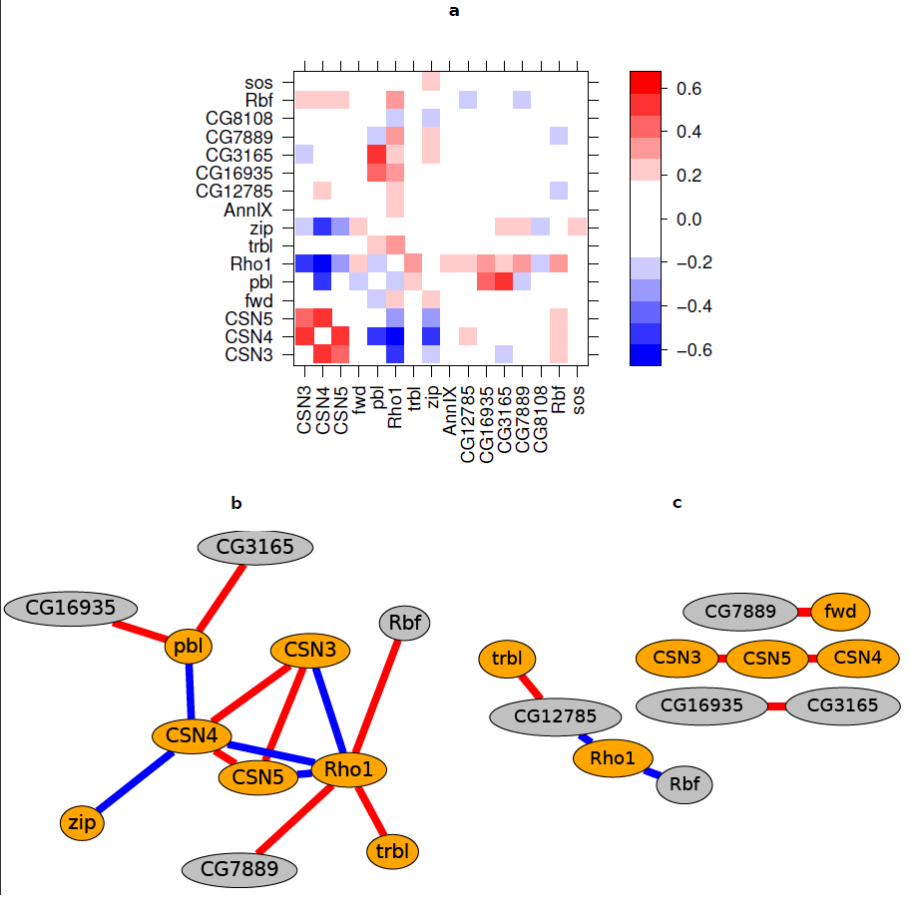
**Interaction matrix and graph**. (a) Heatmap matrix representation of interaction estimates. The colours represent effect size on the log_2 _scale. The eight genes from the cell cycle set, CSN3, CSN4, CSN5, fwd, pbl, Rho1, trbl and zip are shown in the lower and left halves of the matrix. The other eight genes had been selected randomly from the fruit fly genome. (b) Threshold graph representation of the interaction matrix. The edges show all interactions with false discovery rate adjusted [42]*p*-value < 0.1 from the *t*-test and absolute effect size > 0.3. Orange nodes indicate the cell-cycle set, grey nodes the randomly selected set. The colour of an edge represents the sign: positive interactions are red and negative blue. (c) Correlation graph based on the Spearman correlation coefficient. Correlations with absolute correlation coefficient > 0.8 are shown as edges. The colour of an edge represents the sign: positive correlations are red and negative blue.

The detected interactions are summarised in Figure 2. The figure uses three different visualisation tools: the heatmap representation of the interaction matrix ${ŵ}_{ij}$, the threshold graph representation of the same matrix, and the threshold graph representation of the correlation matrix

(7) $$c_{ij}=cor\left({ŵ}_{i}.,{ŵ}_{j}.\right),$$

where ${ŵ}_{i}$ denotes the *i*-th row of the matrix ${ŵ}_{ij}$, i.e. the interaction profile of gene *i *across all other tested genes. Most interactions are seen within the cell-cycle set of genes; there are few interactions between the cell-cycle related genes and the randomly selected set of genes. Among the randomly selected set, Rbf is interacting strongly with Rho1. Rbf is in fact a known cell cycle regulator [34]. The DroID database [35] reported seven interactions between the cell cycle related genes. Of those, three were found significant in our data: CSN4-CSN5 [36], CSN3-CSN4 (predicted) and CSN3-CSN5 (predicted). Among the novel interactions, Rho1 showed negative interactions with CSN3, CSN4 and CSN5 (CSN3-5). Consistent interaction patterns with CSN3-5 are expected as CSN3-5 are three subunits of the COP9 complex.

The directions of the observed interactions are informative: A knock-down of any of the three COP9 subunits resulted in reduced viability. The interactions within CSN3-5 are positive (the negative viability effect of the double knock-down is less severe than the expected effect from the two single knock-downs). One can speculate that knocking down one of the subunits is enough to disrupt the complex and cause a viability effect, and that once the complex is dysfunctional, knocking down another subunit has little additional effect.

The correlation network provides additional insights. It makes evident that the three COP9 subunits have similar interaction profiles and therefore cluster together. An interpretation is that it is the disruption of the COP9 complex, through any of the three subunits, that interacts with the rest of the gene set.

### Scalability - Larger experiments

Future experiments are likely to test a considerably larger number of genes than the experiment discussed here. We analysed a scalability testing (ST) data set, in which a similar experimental design as described above was used to assay cell viability in response to all pairwise interactions of a set of 84 dsRNA reagents. The data preprocessing is shown in Additional File 5: Figure S4 and Additional File 6: Figure S5.

#### Fit diagnostics

**Figure 3:**
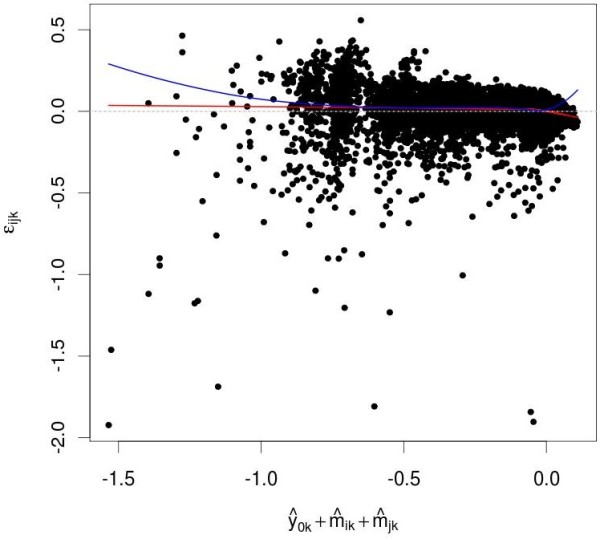
**Fit diagnostics**. ${ŷ}_{0k}+{\hat{m}}_{ik}+{\hat{m}}_{jk}$, the predicted effect of combination (*i*, *j*) if there is no interaction, is plotted along the *x*-axis versus *ε_ijk_*, the difference between observed and predicted value on the *y*-axis. The red line indicate local regression estimates of local mean of *ε_ijk _*[49]. No significant trends are apparent.

Fit diagnostics check how well a data analytical model fits the data by plotting the residual distributions against various explanatory variables. Depending on the viewpoint, they can be used to check the adequacy of a model and to assess the quality of the data. Figure 3 shows the distribution of the residuals *ε_ijk _*against the predicted value ${ŷ}_{0k}+{\hat{m}}_{ik}+{\hat{m}}_{jk}$ for the ST data. Trends in this plot would indicate a model misspecification problem. No significant trends were apparent.

#### False discovery rate

**Figure 4:**
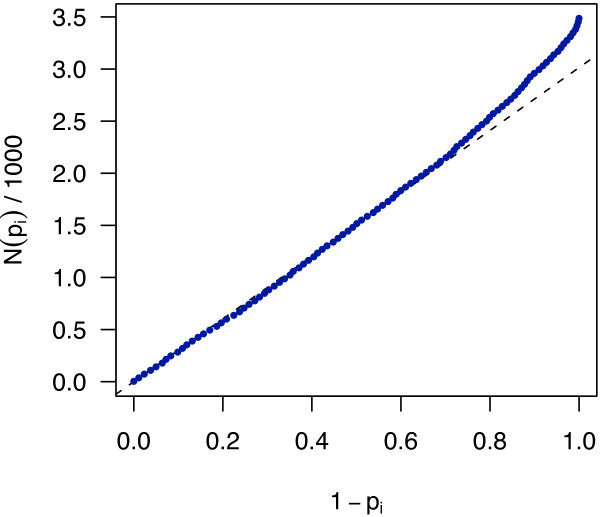
**Schweder and Spjøtvoll plot**. A plot of the *p*-value distribution for the ST data, as described in the text around Equation (8) and as suggested by Schweder and Spjøtvoll [37]. The blue dots correspond to *p*-values from the moderated *t*-test of the *m *= 3486 gene pairs. For visualisation, the graph is represented by 100 points sampled equidistantly along the *x*-axis. The dashed straight line was fit to the values of the graph at *x *= 0 and *x *= 0.5. It intersects the *x *= 1 axis at *y *= 3014, providing an estimate of 3486 - 3014 = 472 false null hypotheses.

Schweder and Spjøtvoll [37] suggested a diagnostic plot of the observed *p*-values that permits estimation of the fraction of true null hypotheses. For a series of *m *hypothesis tests with *p*-values *p_i_*, they suggested plotting

(8) $$\left(1-p_{i},N\left(p_{i}\right)\right)\;for\;i\in 1,\;\ldots ,\;m,$$

where *N*(*p*) is the number of *p*-values greater than *p*. An application of this diagnostic plot to the *p*-values from the moderated *t*-test for the ST data set is shown in Figure 4. If all null hypotheses were true, i.e., no interactions were present, the *p*-values would each be uniformly distributed in [0,1], and the cumulative distribution function of (*p*_1_,..., *p_m_*) would fall close to the line *f*(*x*) = *x*/*m*. In fact, the curve is an approximately straight line with smaller slope within between *x *= 0 and about *x *= 0.5, and then bends upward, indicating that some null hypotheses are not true. The intersection of the dashed line with the vertical axis at *x *= 1 indicates evidence for a number of false null hypotheses around 400 to 500.

### Choice of scale and neutrality function

An analytic expansion like in Equation (2) is always possible, its usefulness, however, and that of the above definition of interactions, depends on the choice of scale of the phenotype variable *y *[23]. Consider, for example, cell number in a cell culture in exponential growth during an incubation time *t*, and suppose that two different genes may be perturbed by RNAi. In the absence of interactions between the two perturbations, we might expect

(9) $$n\left(t\right)=n_{0}e^{\left(1+m_{1}x_{1}+m_{2}x_{2}\right)kt},$$

where *n *is the cell number at time *t*, *n*_0 _is the initial cell number, *k *is the growth rate of the unperturbed cells, the indicator variables *x*_1_, *x*_2 _∈ {0, 1} indicate whether or not the gene was perturbed by RNAi, and *m*_1_, *m*_2 _are the two perturbations' individual effects on growth [38]. If we expand *n *- *n*_0 _directly as in Equation (2), we will get a second-order term between *x*_1 _and *x*_2 _simply due to the presence of the exponential function in Equation (9). However, if we first transform the cell number measurements to a logarithmic scale, for instance, *y *= log(*n*/*n*_wt_), where *n*_wt _= *n*_0 _exp(*kt*) is the cell number at time *t *without perturbation, then *y *is exactly described by the linear expression (*m*_1_*x*_1 _+ *m*_2_*x*_2_) *kt*, which better reflects the fact that the perturbations do not interact. This is the approach we have taken above. In the following, we discuss some points regarding the choice of scale that may need to be considered in different experimental settings.

The exponential growth model (9) may not be appropriate for the whole duration of the experiment. Initially, for small *t*, cells might need some time to recover from an experimental treatment that they were subjected to (such as transfection, seeding) before they go into their optimal growth rate. For larger *t*, cell density might become so large that saturation effects play a role, again decreasing the growth rate.

Furthermore, depending on the measurement setup, in particular for fluorescence or luminescence based measurements, background signal might contribute to the observed data. In the worst case, *k *= *k*(*t*) is a complex time-dependent function, possibly different for different perturbations, and if *n*(*t*) is only observed at one end point *t*, such effects can make it impossible to infer biologically relevant interactions. In some instances, it might be possible to fit a more general growth model (that includes, for instance, an initial lag phase and a background signal).

In some experiments reported in the literature, *biomass production n*(*t*) was monitored over time. This allows measuring the growth rate *n*^-1^(*t*) *dn*(*t*)/*dt *as a function of time. Typically, this function reaches a maximal value at some time between the start and end time of the observation, and this value is used to quantify the growth phenotype. Relative growth rate can be defined as the dimensionless ratio [39]

(10) $$\rho =\frac{max_{t}\frac{dn\left(t\right)}{n\left(t\right)dt}}{max_{t^{\prime }}\frac{dn_{wt}\left(t^{\prime }\right)}{n_{wt}\left(t^{\prime }\right)dt^{\prime }}}.$$

If the perturbation does not affect growth, then *ρ *= 1. If the perturbation promotes growth, *ρ *will be larger than 1, if it is slowing down growth, *ρ *will be less than 1.

Several authors have considered two perturbations to be interacting if the product of their individual relative growth rates, *ρ*_1 _and *ρ*_2_, is different from that of the combinatorial perturbation, i. e. if not *ρ*_12 _= *ρ*_1 _*ρ*_2 _[5,39,40]. This definition of interaction is somewhat different from that implied by (9). In particular, if exponential growth (9) holds, then (10) simplifies to *ρ*_1 _= 1 + *m*_1 _and *ρ*_2 _= 1 + *m*_2_, and according to (9), the two perturbation are considered non-interacting if *ρ*_12 _= 1 + *m*_1 _+ *m*_2 _= *ρ*_1 _+ *ρ*_2 _- 1. For small perturbations, the two definitions are approximately equivalent, since (1 + *m*_1_)(1 + *m*_2_) = 1 + *m*_1 _+ *m*_2 _+ *m*_1_*m*_2 _and *m*_1_*m*_2 _is negligible if *m*_1 _and *m*_2 _are small compared to 1; for instance, for *m*_1 _= *m*_2 _= 0.1, *m*_1_*m*_2 _= 0.01.

For strong perturbations, however, they lead to different results. For the present work, we chose (9) because it allows a more coherent treatment of data in which *dn*(*t*)/*dt *is positive for some of the cases (typically, for the wild type) and negative for others (say, for RNAi that knocks down an apoptosis inhibitor). In those cases, *ρ *can become negative, and interpretation of the multiplicative neutrality function *ρ*_12 _= *ρ*_1 _*ρ*_2 _would be difficult.

## Conclusions

We have discussed all steps in the data analysis of combinatorial RNAi screens, from experimental design to hit list, including adjustment for plate effects and data quality assessment, estimation of main effects and interactions, fit diagnostics, assessing statistical significance, visualisation and exploration of the interaction network.

### Assessing significance

To assess significance of interactions, we used the moderated *t*-test. Compared to the ordinary *t*-test, it provides much better detection power when the number of replicates is small.

However, there are caveats with *t*-test approaches, and more sophisticated criteria might be needed for some applications. The first caveat pertains to the fact that the *t*-statistic compares average effect size to the estimated standard deviation of the effects. Hence it penalises interactions that are strong, but whose quantitative effect is highly variable between replicates; if such penalisation is desired, a *t*-statistic is appropriate, but in some cases, such interactions may be of interest.

The second caveat is that the null hypothesis of the *t*-test - that the effect size is *exactly *zero - is unrealistic, and with more and more data, more and more null hypotheses will be rejected, resulting in many called interactions that are statistically significant, but of very small size. This problem, which can be illustrated by Lindley's paradox [41], is a well known shortcoming of simple hypothesis testing. In this paper, we considered a data set that had 20 replicate measurements for each pair of genes (Figure 2), and a large number of gene pairs had a small *p*-value. In Figure 2, we only show those edges with *t*-test, false discovery rate adjusted [42]*p*-values below 0.1 *and *with an absolute average effect size greater than 0.3. Setting such an effect size cutoff may appear somewhat ad hoc, and more theoretically founded statistical techniques exist: in a Bayesian approach, a prior can be used that encodes a preference for finding many gene pairs without interactions; similarly, a joint, sparse regression model could be used, using penalisation on interaction efficients, as with LASSO [43].

In the ST data, the number of replicates tested per gene pair was much smaller, and the problem of many statistically significant, but small interactions did not arise. However, when we computed the correlation threshold graph, such as in Figure 2c, from correlation tests between all pairs of interaction profiles, a similar problem arose: the correlation test has a sample size of 84, and hence is able to pick up weak, but statistically significant correlations between gene interaction profiles. Again, in addition to statistical significance, we used a threshold on the absolute correlation coefficient in order to focus on the larger effects.

### Interaction correlation networks

Individual genetic interactions can be biologically interesting and relevant. This was illustrated by the individual interactions between the three COP9 complex homolog subunits, CSN3-5. However, individual interactions do not generally seem to be well-conserved across species [44] and even within species, may depend on genetic background, cell type and environmental conditions. A property of gene pairs that appears to be more generally conserved, and in many cases capable of producing more robust insights, is the (dis)similarity of their interaction profiles [9].

### Other phenotypes

Our treatment was designed for *cell growth *phenotypes. New technologies, in particular microscopy, are now delivering measurements of many other kinds of phenotypes, such as delays in cell cycle timing or changes to cells' morphology and motility [45-47]. How to detect, and in fact, even to define what are, genetic interactions for such phenotypes remains an open question, and an exciting topic for future research.

## Availability and requirements

A Bioconductor package, *coRNAi*, implementing our methodology and containing all code to reproduce the results presented here, is freely available from Bioconductor [48]http://www.bioconductor.org/packages/release/bioc/html/coRNAi.html.

## Competing interests

The authors declare that they have no competing interests.

## Authors' contributions

All authors designed the research, TH and TS performed the experiments, EA performed the data analysis and methods development, with input from all authors, EA and WH wrote the article with input from all authors. All authors have read and approved the final manuscript.

## Supplementary Material

Additional file 1 **Table S1 - Gene ontology (GO) annotation terms**. Click here for file

Additional file 2 **Figure S1 - Per plate boxplots**. The distributions of the logarithm-transformed intensities varied over different plates. (a) After centering, the locations were the same (horizontal bars within boxes). (b) Shown are the data from sample (i. e. non-control) wells only. Plates 1-5 are technical replicates of the first biological replicate, plates 6-10 are technical replicates of the second biological replicate. Click here for file

Additional file 3 **Figure S2 - Spatial patterns**. False colour representation of the spatial pattern of the intensities after normalisation, on the same log_2_-transformed scale as in Figure Additional File 2: Figure S1b. Each plate contained 384 (16 times 24) wells. On every plate, the positive controls, shown in dark red, are in columns 7 and 14. Plates 1-5 (top row) are technical replicates of the first biological replicate, plates 6-10 (bottom row) are technical replicates of the second biological replicate. This structure of the experimental design is reflected in somewhat different dynamic ranges (sizes of the strongest positive and negative effects) between the biological replicates. Overall, the plots indicate that the spatial patterns seen are consistent with expected biological effects and show no evidence of xy position-dependent artifacts [28]. Click here for file

Additional file 4 **Figure S3 - Replicate reproducibility**. Two technical replicates, plotted against each other in the scatter plot, showed high correlation and no outliers. Shown are the data from sample (i. e. non-control) wells only. Similar reproducibility was seen for all technical replicates. Click here for file

Additional file 5 **Figure S4 - Per plate boxplots for the ST data**. The ST data were measured on 24 plates. The distributions of the logarithm-transformed intensities varied over the plates (a). After centering, the locations were the same (horizontal bars within boxes) (b). Shown are the data from sample (i. e. non-control) wells only. Click here for file

Additional file 6 **Figure S5 - Quality assessment for the ST data**. Panel (a) shows a false colour representation of the spatial pattern of the intensities after normalisation, on a log_2_-transformed scale, across the 24 plates of the ST data. Each plate contained 16 times 24 wells. On every plate, the positive controls, shown dark red, are in the rightmost column and in the second row from the bottom, except for well O18. The spatial patterns seen are consistent with expected biological effects and show no evidence of artifacts. Panels (b) and (c) show diagnostics of the separation of the positive and negative controls. Panel (b) shows, for each plate along the *x*-axis, the values of positive (red) and negative (blue) controls along the *y*-axis. The same data is also shown in the density plot in Panel (c). Panels (b) and (c) as well as the *Z*'-factor = 0.86 [50] indicate good separation between the positive and negative controls throughout the screen. Click here for file
