# Supplementary material for: Extracting quantitative genetic interaction phenotypes from matrix combinatorial RNAi
Source: BMC Bioinformatics. 2011 Aug 17;12:342. doi: 10.1186/1471-2105-12-342 (PMC3230910; doi:10.1186/1471-2105-12-342)
Supplement: Additional file 1 — Table S1 - Gene ontology (GO) annotation terms. [file 1471-2105-12-342-S1.PDF]

Table S.1: The 8 cell-cycle related genes with associated GO terms followed by the 8 randomly selected genes with associated GO terms (when available)

Gene ID	Accession	GO term
CSN3	GO:0007095	mitotic cell cycle G2/M transition DNA damage checkpoint
	GO:0050821	protein stabilization
CSN4	GO:0001751	compound eye photoreceptor cell differentiation
	GO:0007095	mitotic cell cycle G2/M transition DNA damage checkpoint
	GO:0032435	negative regulation of proteasomal ubiquitin-dependent protein catabolic process
	GO:0000338	protein deneddylation
	GO:0050821	protein stabilization
CSN5	GO:0001751	compound eye photoreceptor cell differentiation
	GO:0008347	glial cell migration
	GO:0007095	mitotic cell cycle G2/M transition DNA damage checkpoint
	GO:0007275	multicellular organismal development
	GO:0032435	negative regulation of proteasomal ubiquitin-dependent protein catabolic process
	GO:0007314	oocyte anterior/posterior axis specification
	GO:0007310	oocyte dorsal/ventral axis specification
	GO:0048477	oogenesis
	GO:0045787	positive regulation of cell cycle
	GO:0000338	protein deneddylation
	GO:0050821	protein stabilization
fwd	GO:0000916	contractile ring contraction involved in cell cycle cytokinesis
	GO:0007110	cytokinesis after meiosis I
	GO:0007111	cytokinesis after meiosis II
	GO:0008340	determination of adult lifespan
	GO:0030726	male germline ring canal formation
	GO:0007112	male meiosis cytokinesis
	GO:0043147	meiotic spindle stabilization
	GO:0016310	phosphorylation
	GO:0048137	spermatocyte division
	GO:0007283	spermatogenesis
pbl	GO:0007155	cell adhesion
	GO:0000910	cytokinesis
	GO:0007110	cytokinesis after meiosis I
	GO:0007111	cytokinesis after meiosis II
	GO:0000915	cytokinesis, actomyosin contractile ring assembly
	GO:0045184	establishment of protein localization
	GO:0007443	Malpighian tubule morphogenesis
	GO:0007509	mesoderm migration involved in gastrulation
	GO:0008078	mesodermal cell migration
	GO:0007399	nervous system development

	GO:0007422	peripheral nervous system development
	GO:0050770	regulation of axonogenesis
	GO:0008360	regulation of cell shape
	GO:0051225	spindle assembly
Rho1	GO:0030036	actin cytoskeleton organization
	GO:0051017	actin filament bundle assembly
	GO:0007015	actin filament organization
	GO:0007411	axon guidance
	GO:0007298	border follicle cell migration
	GO:0035147	branch fusion, open tracheal system
	GO:0090254	cell elongation involved in imaginal disc-derived wing morphogenesis
	GO:0007349	cellularization
	GO:0001745	compound eye morphogenesis
	GO:0000910	cytokinesis
	GO:0007010	cytoskeleton organization
	GO:0048813	dendrite morphogenesis
	GO:0007368	determination of left/right symmetry
	GO:0007391	dorsal closure
	GO:0046663	dorsal closure, leading edge cell differentiation
	GO:0007395	dorsal closure, spreading of leading edge cells
	GO:0006897	endocytosis
	GO:0007173	epidermal growth factor receptor signaling pathway
	GO:0001737	establishment of imaginal disc-derived wing hair orientation
	GO:0001736	establishment of planar polarity
	GO:0045184	establishment of protein localization
	GO:0007164	establishment of tissue polarity
	GO:0007369	gastrulation
	GO:0010004	gastrulation involving germ band extension
	GO:0008354	germ cell migration
	GO:0007377	germ-band extension
	GO:0008347	glial cell migration
	GO:0035099	hemocyte migration
	GO:0035317	imaginal disc-derived wing hair organization
	GO:0007254	JNK cascade
	GO:0035149	lumen formation, open tracheal system
	GO:0045199	maintenance of epithelial cell apical/basal polarity
	GO:0035006	melanization defense response
	GO:0008045	motor axon guidance
	GO:0007405	neuroblast proliferation
	GO:0016318	ommatidial rotation
	GO:0007424	open tracheal system development
	GO:0007422	peripheral nervous system development
	GO:0007374	posterior midgut invagination
	GO:0030589	pseudocleavage involved in syncytial blastoderm formation

	GO:0050770	regulation of axonogenesis
	GO:0051493	regulation of cytoskeleton organization
	GO:0016476	regulation of embryonic cell shape
	GO:0035298	regulation of Malpighian tubule size
	GO:0035159	regulation of tube length, open tracheal system
	GO:0006974	response to DNA damage stimulus
	GO:0007435	salivary gland morphogenesis
	GO:0035277	spiracle morphogenesis, open tracheal system
	GO:0007370	ventral furrow formation
	GO:0016055	Wnt receptor signaling pathway
	GO:0042060	wound healing
trbl	GO:0007369	gastrulation
	GO:0045839	negative regulation of mitosis
	GO:0051726	regulation of cell cycle
	GO:0007370	ventral furrow formation
zip	GO:0009653	anatomical structure morphogenesis
	GO:0035017	cuticle pattern formation
	GO:0000910	cytokinesis
	GO:0007391	dorsal closure
	GO:0046664	dorsal closure, amnioserosa morphology change
	GO:0046663	dorsal closure, leading edge cell differentiation
	GO:0007395	dorsal closure, spreading of leading edge cells
	GO:0035072	ecdysone-mediated induction of salivary gland cell autophagic cell death
	GO:0045200	establishment of neuroblast polarity
	GO:0001736	establishment of planar polarity
	GO:0045184	establishment of protein localization
	GO:0008258	head involution
	GO:0035317	imaginal disc-derived wing hair organization
	GO:0007443	Malpighian tubule morphogenesis
	GO:0006936	muscle contraction
	GO:0030239	myofibril assembly
	GO:0031036	myosin II filament assembly
	GO:0007297	ovarian follicle cell migration
	GO:0051259	protein oligomerization
	GO:0035159	regulation of tube length, open tracheal system
	GO:0007435	salivary gland morphogenesis
	GO:0045214	sarcomere organization
AnnIX	GO:0048190	wing disc dorsal/ventral pattern formation
CG12785	NA	NA
CG16935	GO:0006631	fatty acid metabolic process
CG7889	NA	NA
CG8108	NA	NA
Rbf	GO:0042023	DNA endoreduplication
	GO:0007307	eggshell chorion gene amplification

	GO:0007113	endomitotic cell cycle
	GO:0000080	G1 phase of mitotic cell cycle
	GO:0007095	mitotic cell cycle G2/M transition DNA damage checkpoint
	GO:0043066	negative regulation of apoptosis
	GO:0008285	negative regulation of cell proliferation
	GO:0008156	negative regulation of DNA replication
	GO:0010629	negative regulation of gene expression
	GO:0045749	negative regulation of S phase of mitotic cell cycle
	GO:0000122	negative regulation of transcription from RNA polymerase II promoter
	GO:0051726	regulation of cell cycle
	GO:0051101	regulation of DNA binding
sos	GO:0007015	actin filament organization
	GO:0008595	anterior/posterior axis specification, embryo
	GO:0045749	negative regulation of S phase of mitotic cell cycle
	GO:0007399	nervous system development
	GO:0007265	Ras protein signal transduction
	GO:0008360	regulation of cell shape
	GO:0045500	sevenless signaling pathway
	GO:0008293	torso signaling pathway
	GO:0007426	tracheal outgrowth, open tracheal system
