# Supplementary figures and images for: Extracting quantitative genetic interaction phenotypes from matrix combinatorial RNAi
Source: BMC Bioinformatics. 2011 Aug 17;12:342. doi: 10.1186/1471-2105-12-342 (PMC3230910; doi:10.1186/1471-2105-12-342)

**a**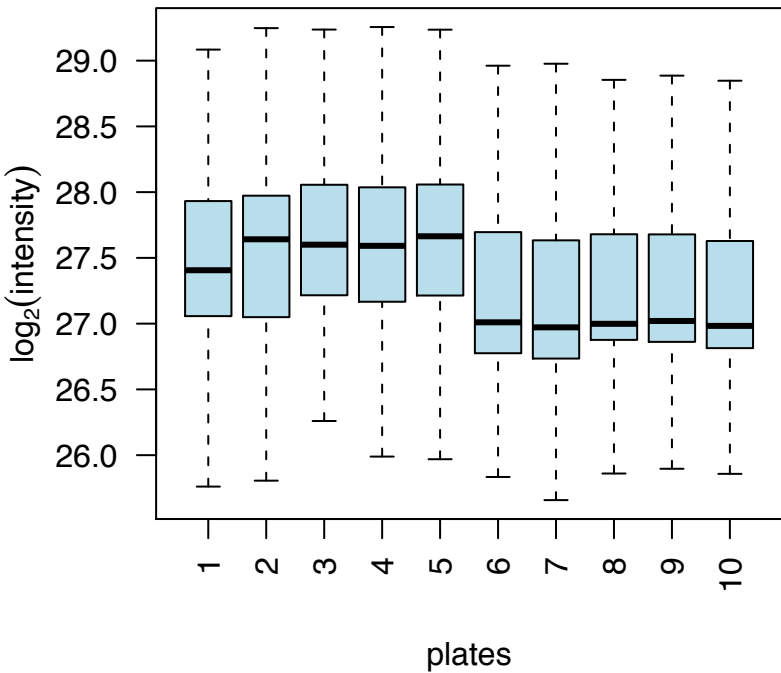**b**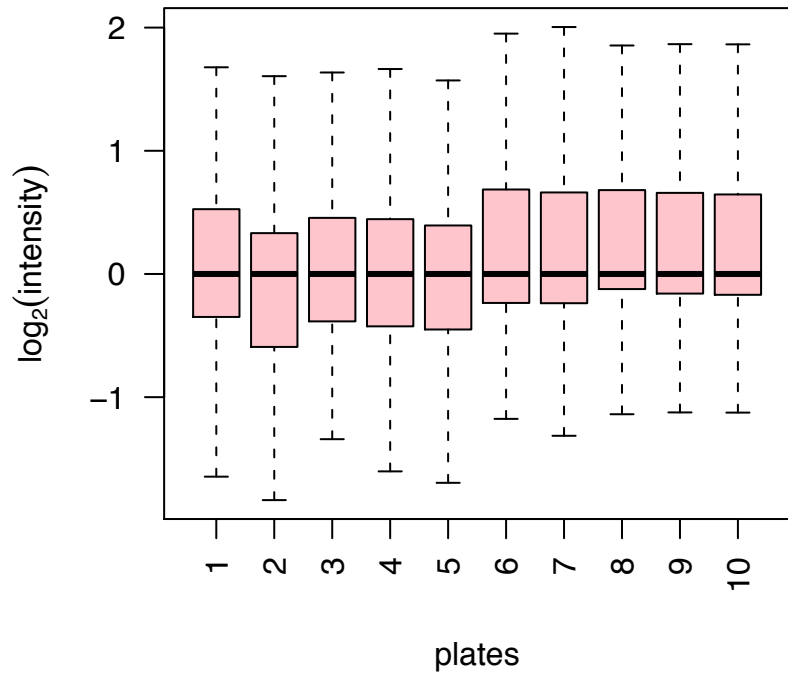

Supplement: Additional file 2 — Figure S1 - Per plate boxplots. The distributions of the logarithm-transformed intensities varied over different plates. (a) After centering, the locations were the same (horizontal bars within boxes). (b) Shown are the data from sample (i. e. non-control) wells only. Plates 1-5 are technical replicates of the first biological replicate, plates 6-10 are technical replicates of the second biological replicate. [file 1471-2105-12-342-S2.PDF]

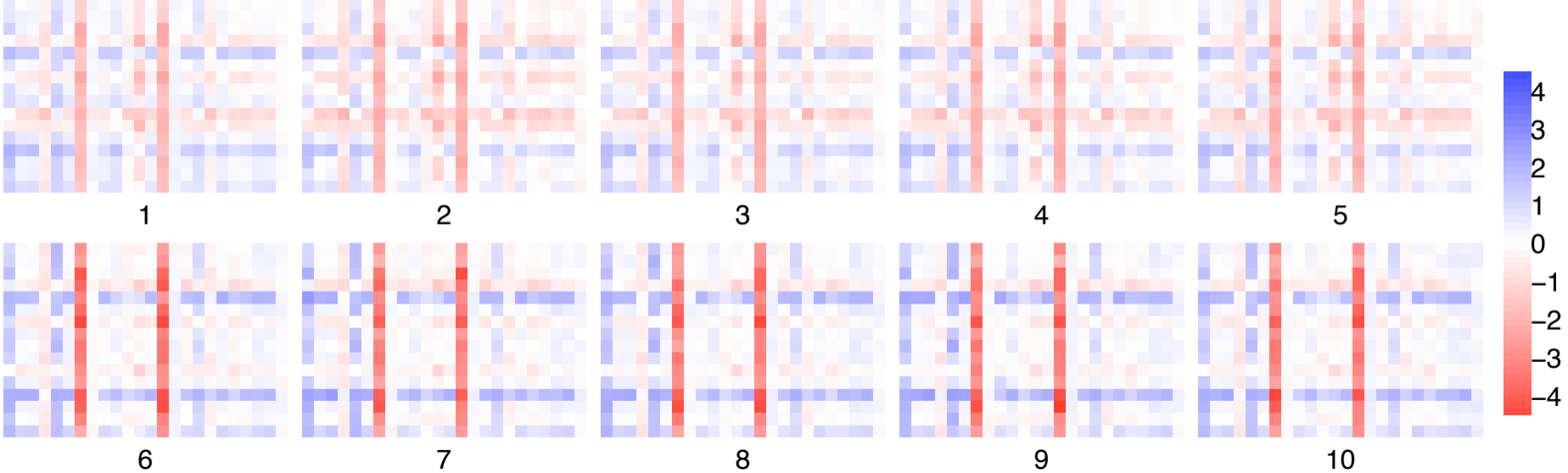

Supplement: Additional file 3 — Figure S2 - Spatial patterns. False colour representation of the spatial pattern of the intensities after normalisation, on the same log2-transformed scale as in Figure Additional File 2: Figure S1b. Each plate contained 384 (16 times 24) wells. On every plate, the positive controls, shown in dark red, are in columns 7 and 14. Plates 1-5 (top row) are technical replicates of the first biological replicate, plates 6-10 (bottom row) are technical replicates of the second biological replicate. This structure of the experimental design is reflected in somewhat different dynamic ranges (sizes of the strongest positive and negative effects) between the biological replicates. Overall, the plots indicate that the spatial patterns seen are consistent with expected biological effects and show no evidence of xy position-dependent artifacts [28]. [file 1471-2105-12-342-S3.PDF]

technical replicate 2

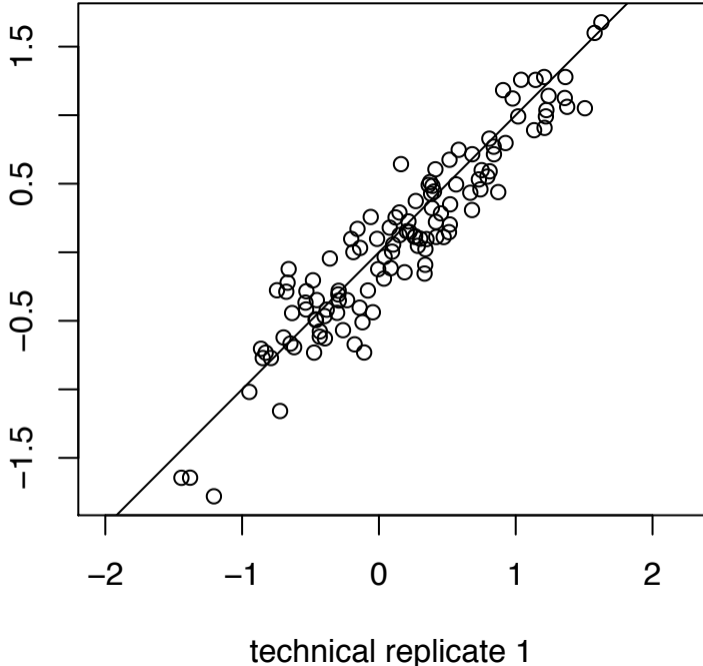

technical replicate 1

Supplement: Additional file 4 — Figure S3 - Replicate reproducibility. Two technical replicates, plotted against each other in the scatter plot, showed high correlation and no outliers. Shown are the data from sample (i. e. non-control) wells only. Similar reproducibility was seen for all technical replicates. [file 1471-2105-12-342-S4.PDF]

**a**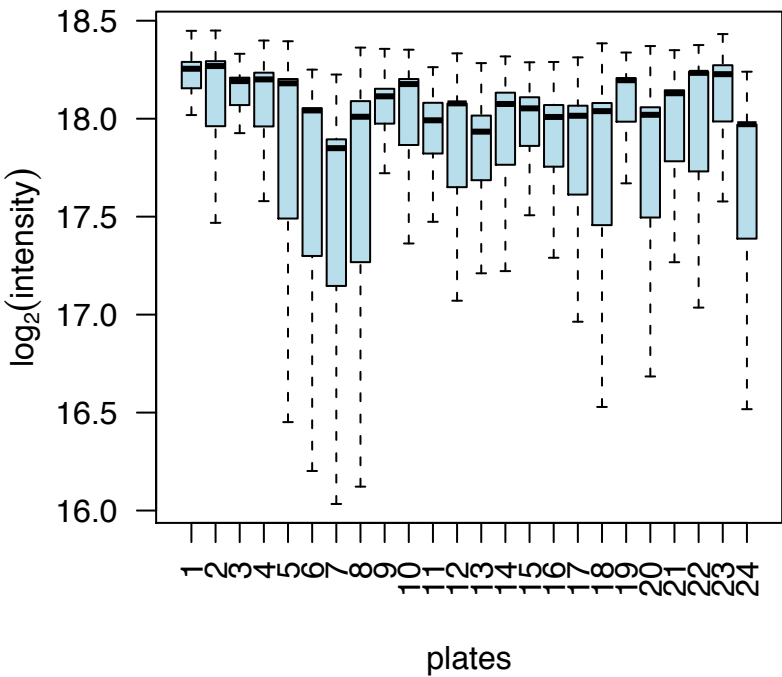**b**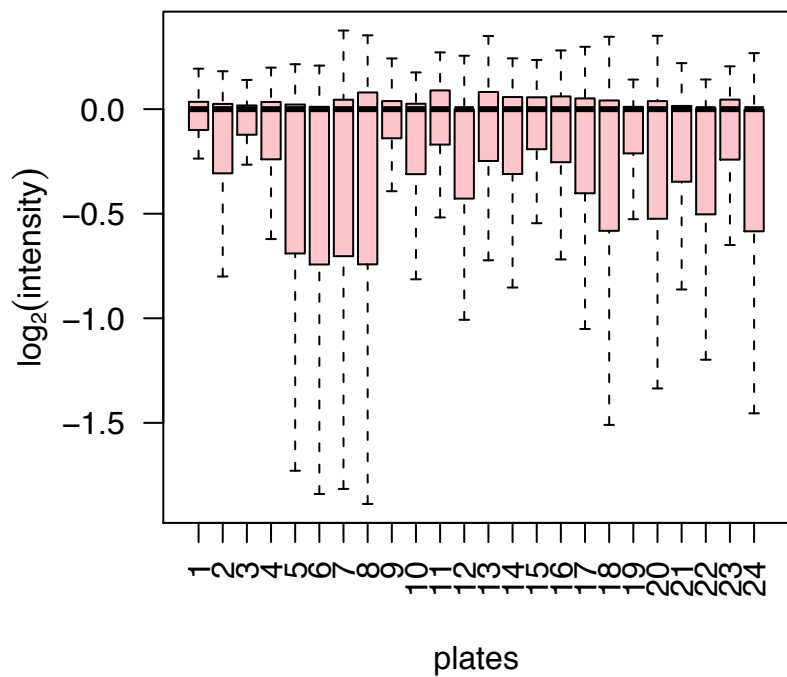

Supplement: Additional file 5 — Figure S4 - Per plate boxplots for the ST data. The ST data were measured on 24 plates. The distributions of the logarithm-transformed intensities varied over the plates (a). After centering, the locations were the same (horizontal bars within boxes) (b). Shown are the data from sample (i. e. non-control) wells only. [file 1471-2105-12-342-S5.PDF]

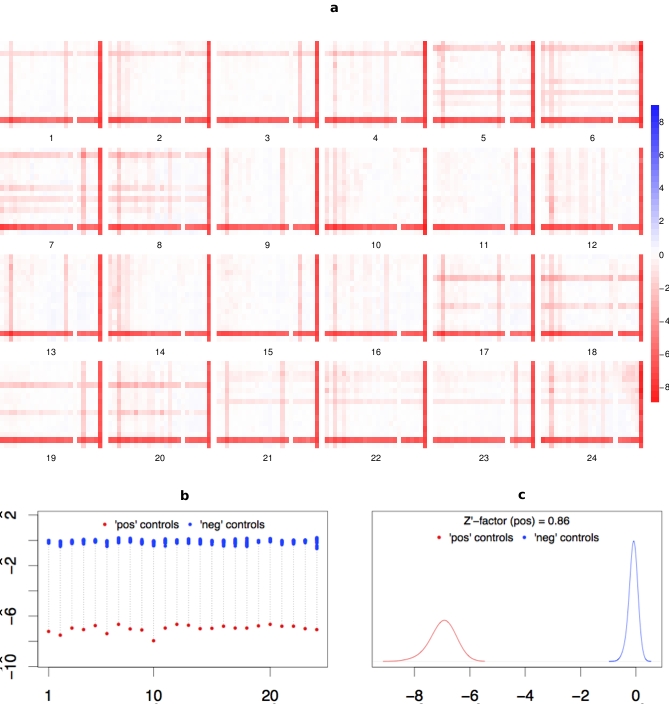

Supplement: Additional file 6 — Figure S5 - Quality assessment for the ST data. Panel (a) shows a false colour representation of the spatial pattern of the intensities after normalisation, on a log2-transformed scale, across the 24 plates of the ST data. Each plate contained 16 times 24 wells. On every plate, the positive controls, shown dark red, are in the rightmost column and in the second row from the bottom, except for well O18. The spatial patterns seen are consistent with expected biological effects and show no evidence of artifacts. Panels (b) and (c) show diagnostics of the separation of the positive and negative controls. Panel (b) shows, for each plate along the x-axis, the values of positive (red) and negative (blue) controls along the y-axis. The same data is also shown in the density plot in Panel (c). Panels (b) and (c) as well as the Z'-factor = 0.86 [50] indicate good separation between the positive and negative controls throughout the screen. [file 1471-2105-12-342-S6.JPEG]
